# Research on the Influence of Labor Contract on the Urban Integration of Migrant Workers: Empirical Analysis Based on China’s Micro Data

**DOI:** 10.3390/ijerph191811604

**Published:** 2022-09-15

**Authors:** Chuangxin Zhao, Manping Tang

**Affiliations:** College of Management, Sichuan Agricultural University, Chengdu 611130, China

**Keywords:** labor contract, urban integration, migrant workers, social security, China

## Abstract

Using the micro data of the China Labor Dynamics Survey (CLDS), this paper uses factor analysis to construct urban integration indicators and uses the OLS model and intermediary effect model to study the urban integration of 1976 migrant workers in 29 cities in China. This paper empirically analyzes the impact of labor contracts on migrant workers’ urban integration and its mechanism. The study found that: (1) labor contract can significantly promote the urban integration of migrant workers. Further, this conclusion is still tenable after correcting endogenous bias with the 2SLS model and performing a series of robustness tests. (2) Signing labor contracts increases the participation rate of migrant workers in various insurances, enhances the social security level of migrant workers, alleviates the discrimination in the urban labor market, and thus enhances the urban integration of migrant workers. (3) The results of the heterogeneity tests show that the labor contract has a greater impact on the urban integration of the new generation, married and public sector of migrant workers compared with the old generation, unmarried and private sector of migrant workers. Therefore, this paper makes the following suggestions: the government should strengthen labor market supervision, encourage employers to sign long-term labor contracts with migrant workers, improve the social security system for migrant workers, and protect the legitimate rights and interests of migrant workers.

## 1. Introduction

Migrant workers are a new type of labor force that has grown rapidly along with industrialization and urbanization since China’s reform and opening up [1,2]. According to the Migrant Workers Monitoring and Survey Report released by the National Bureau of Statistics, the total number of migrant workers in China in 2021 was 292.51 million, an increase of 6.91 million over to the previous year, including 171.72 million migrant workers who worked in an urban area, an increase of 2.13 million over the previous year [3]. Migrant workers are an important group that cannot be ignored in China’s social development, and who have engaged in various industries and have made great contributions to China’s urbanization and social and economic development [4]. However, a large number of migrant workers are unable to integrate into cities, do not enjoy the same benefits and treatment as urban residents, and are always affected by household registration and geographical discrimination [5]. There exists the phenomenon of “different pay and different rights for the same work” between migrant workers and urban labor forces from the perspective of employment. Meanwhile, the difficulty of urban integration of migrant workers is also an important reason for many unbalanced problems due to the existence of the urban-rural dual system [6]; in addition, the limited education of children also hinders the urban integration of migrant workers to a certain extent [7]. The experience of world economic development shows that urbanization is the only way for modernization and a powerful driving force for sustainable economic development [8]. To some extent, only by solving the problem of urban integration of migrant workers can we further realize people-oriented urbanization [9,10]. Therefore, in the context of the new era, it is of great significance to transform migrant workers’ status into citizen, get rid of urban marginalization, and promote the integration of migrant workers into the city so as to improve their welfare as well as happiness index, and promote urbanization and socioeconomic development.

The labor contract is an important legal basis for safeguarding the legal rights and interests of migrant workers, and is the cornerstone of ensuring that migrant workers enjoy labor protection, social insurance, wages and benefits and other rights and benefits. The Chinese government officially implemented the “Labor Contract Law” in 2008, which included migrant workers and other flexible employment workers into the scope of protection, aiming to protect the legitimate rights and interests of low-skilled workers. Since the implementation of the Labor Contract Law, the proportion of migrant workers signing labor contracts has increased year by year, effectively improving the labor welfare of migrant workers [11]. Exploring the impact of labor contracts on the urban integration of migrant workers is a beneficial extension of migrant workers’ research, and it can also provide policy implications for promoting the development of new people-oriented urbanization.

Urban integration refers to the process in which the floating population adapts to urban residents, urban life, and urban culture on the basis of enjoying various basic rights, and gradually reduces the gap and differences with urban residents in this process. Generally speaking, the main concepts used in the West about the integration of floating population are social adaptation, social absorption, assimilation, and social integration, etc. [12,13], and scholars have tried to study the social integration of migrants in the workplace from different perspectives and levels. The research on social integration mainly focuses on the urban integration of migrant workers in China. A lot of studies have been done on the social integration of the floating population at home and abroad. Social integration is a multi-dimensional indicator, and Gordon [14] proposed the so-called “two-dimensional” division method, which includes cultural and structural dimensions. Junger-Tas et al. [15] believed that the social integration of immigrants should include structural integration, social and cultural integration, and political legitimacy integration, which was the famous “three-dimensional” model. Entzinger et al. [16] further developed the previous studies and proposed a four-dimensional model, which summarized the social integration of immigrants into four dimensions: socioeconomic integration, political integration, cultural integration, and the social acceptance or rejection of immigrants. Yang [17] argued that social integration includes four dimensions: economic integration, cultural acceptance, behavioral adaptation, and identity. Wang et al. [18] constructed an evaluation index system of social integration, which includes five first-level indicators of living conditions, social relations, political participation, economic life and psychological identity. Zhang et al. [19] measured the degree of social integration of relocated households through four dimensions: economic integration, psychological integration, identity integration, and community and cultural integration. Generally speaking, scholars generally agree that the dimensions of social integration mainly includes five dimensions: economy, society, culture, psychology, and identity [20].

There are abundant studies on the influencing factors of the urban integration of migrant workers, and academic circles have mainly summarized the influencing factors of urban integration of migrant workers into economic factors, human capital factors, social capital factors, and institutional capital factors [21,22,23,24]. Stable economic income is the foundation for migrant workers to live in cities, and economic factors mainly include income and expenditure [25], employment stability, housing security, and urban housing prices [26]. Human capital factors mainly emphasize the human capital characteristics of individuals, such as educational background, vocational training, work experience, and language skills, etc. [27]. Takeda et al. [28] found that if immigrants are in a perfectly competitive market in the immigrant region, the human capital level directly determines how much economic success immigrants can achieve. Social capital factors raise the level and angle of analysis to the level of social relations and social networks of individuals compared with human capital factors, and social capital factors include social relations, social networks, and social resources, etc. [29]. The social capital and social networks of migrant workers play a role in providing information, social support, and emotional support [30]. Institutional factors emphasize the restrictive effect of institutional policies on the urban integration of migrant workers [31], including systems and policies in social welfare and social security, social discrimination, religious belief, children’s education, and social assistance [32,33]. In China, the household registration system is the main institutional factor affecting the urban integration of migrant workers, and the household registration system may aggravate the contradiction between urban and rural areas in the process of urbanization in China, thus affecting the quality of urban economic development [34].

Others have done a lot of research on the urban integration of migrant workers, but there is less research on the relationship between the labor contract and the urban integration of migrant workers, and there are still a lot of problems to be further studied. The question, then, becomes whether the labor contract has an effect on the urban integration of migrant workers. Are there group differences in this effect? Furthermore, how does the labor contract affect the urban integration of migrant workers? In order to answer the above questions, this paper uses factor analysis to construct a comprehensive index of migrant workers’ urban integration based on the 2018 China Labor Dynamics Survey data, and uses an ordinary least squares and intermediary effect model to empirically analyze the impact of the labor contract on migrant workers’ urban integration and its mechanism. This study hopes to expand and enrich the research system of migrant workers’ urban integration and provide experience, support, and policy reference for promoting the new urbanization strategy and promoting urban-rural integration.

The main contributions of this paper are as follows: On the one hand, from the perspective of research, taking social security as the starting point, it focuses on discussing the important influencing factor that has been neglected in the research on migrant workers’ urban integration—the labor contract. On the other hand, in terms of research content, this paper not only discusses the impact of labor contracts on migrant workers’ urban integration, but also analyzes the heterogeneity of age, marital status, and the nature of work units. In addition, this study also discusses and tests the impact mechanisms, further deepening the knowledge and understanding of the relationship between the labor contract and the urban integration of migrant workers.

## 2. Theoretical Analysis

Drawing on existing research, this paper divides urban integration into three levels: economic integration, social adaptation, and psychological integration. Although there are few studies directly related to labor contracts and the urban integration of migrant workers, the main logical chains of the relationship between labor contracts and the urban integration of migrant workers can be roughly sorted out from the existing literature. First, labor contracts may affect the economic integration of migrant workers in the inflow area. Economic integration mainly emphasizes the employment opportunities, wages, social security, occupational prestige, vocational training and working environment of migrant workers in the labor market [35]. Signing the labor contract can systematically protect the legal rights and interests of migrant workers, ensure that migrant workers enjoy corresponding rights and interests in terms of wages, vocational training, and social security, reduce the risk of migrant workers being dismissed, and establish stable labor relationships, which will help to improve the job satisfaction of migrant workers and realize self-worth, and thus promote the integration of migrant workers into cities. Second, labor contracts may affect the social adaptation of migrant workers in the inflow area. According to Maslow’s hierarchy of needs theory, social interaction and respect are the basic needs of human beings, and only by establishing harmonious, intimate, trusting and mutually helpful relationships with others can we get support and recognition from others, thereby enhancing life happiness. Signing the labor contract enables migrant workers to become formal employees of the unit, and allows them to join and participate in various activities organized by the labor union, which is conducive to expanding the circle of friends and social networks [36]. Migrant workers can obtain trust support, information support and membership support from social networks, which can help migrant workers cope with problems and difficulties in life and work, improve the ability to adapt to urban life, and thus promote urban social integration [37]. Finally, labor contracts may affect the psychological integration of migrant workers in inflow areas. Psychological integration refers to the psychological distance between migrant workers and residents in the inflow area [17]. The signing of labor contracts between units and migrant workers is a kind of identity recognition for migrant workers, which can draw the psychological distance between migrant workers and local people, and give the sense of belonging of migrant workers, and thus promote the integration of migrant workers into cities. Thus, this paper proposes Hypothesis 1:

**Hypothesis** **1.** *Signing labor contracts has a positive effect on the urban integration of migrant workers*.

Migrant workers face significant employment discrimination in the urban labor market due to the existence of the urban-rural dual household registration system. Migrant workers are subject to household registration discrimination in terms of wage income, pension insurance, medical insurance and union involvement in addition to the labor contract [38]. Compared with local urban workers, migrant workers have suffered from 26% regional discrimination and 30.5% household registration discrimination [39]. The household registration system restricts migrant workers’ access to some basic public services and weakens the sense of identity and belonging to urban life, thus hindering the urban integration of migrant workers [40]. The labor contract system is an important entry point for local labor and foreign labor to enjoy equal social welfare. On the one hand, signing labor contracts can significantly increase the wage level and social insurance participation rate of migrant workers. Signing labor contracts increases the probability of migrant workers participating in endowment insurance and medical insurance by six times compared with who did not sign labor contracts, and signing long-term labor contracts is more conducive to improving the social insurance participation rate than signing short-term labor contracts [41]. Since the implementation of the new “Labor Contract Law” in China, the proportion of migrant workers with social insurances has increased by 10–26% [42], and the social security level of migrant workers has been effectively improved. On the other hand, from the perspective of social security theory, the social insurance system acts as a risk dispersion system which can effectively reduce the uncertain risk of the future development of migrant workers and enhance the confidence of migrant workers in future income [42], which is conducive to improving the social welfare level and quality of life of migrant workers, thereby increasing migrant workers’ willingness to settle in urban areas. In view of this, this paper puts forward the following research hypothesis.

**Hypothesis** **2.** *Social security plays a mediating role in the relationship between the labor contract and urban integration of migrant workers*.

## 3. Materials and Methodology

### 3.1. Data Sources

The data used in this paper come from the China Labor Force Dynamics Survey (CLDS) in 2018 organized by the Science Survey Center of Sun Yat-sen University. The survey covers 29 provinces and cities in China (except Hong Kong, Macao, Taiwan, Tibet, and Hainan). A total of 16,537 individual questionnaires of the labor force population are surveyed from 13,501 households in 368 communities by using a scientific sampling design with multiple stages and levels proportional to the size of the labor force, and the survey respondents are laborers aged 15–64. The content focuses on the current situation of, and changes in, educational experience, work status, entrepreneurial process, social participation and social integration, economic activities, labor status and other information. It has strong representativeness and provides a good data source for this study. In this paper, the population with agricultural household registration and earning wage income in CLDS is counted as migrant workers (This is different from the definition of migrant workers by China’s National Bureau of Statistics (NBS), which defines migrant workers as those whose household registration is still in rural areas and who are engaged in non-agricultural industries locally or have been working outside for six months or more). A total of 1976 valid samples were obtained after excluding the missing samples of the main variables such as the labor contract and social insurance participation.

### 3.2. Variable Selection

#### 3.2.1. Explained Variable

The explained variable of this paper is the urban integration of migrant workers. It is necessary to select appropriate indicators to measure each dimension after determining the measurement dimensions of urban integration. Economic integration is the survival basis for migrant workers to integrate into cities, and only with relatively stable jobs and income in cities can migrant workers gain a long-term foothold in cities [43]. This paper measures the economic integration degree of migrant workers from five aspects: salary income, job satisfaction, satisfaction with respect for work, work environment satisfaction and household consumption expenditure. Participating in the decision-making and discussion of public affairs in the community and gaining a sense of participation is the premise of social adaptation, which focuses on social communication and social interaction between migrant workers and indigenous residents [44]. This paper mainly measures the social adaptation of migrant workers from five aspects: participating in village/neighborhood committee elections, familiarity with local residents, mutual assistance with local residents, level of trust in local residents, and local life happiness. Psychological integration is a higher level of urban integration of migrant workers [17] which is manifested in the identification of values and lifestyles. Better psychological integration is related to the number of friends that migrant workers have close relationships with in the local area. Discussing important issues and sharing the concerns with friends is an important aspect to relieve psychological pressure and adapt to local life. In view of this, this paper measures the psychological integration of migrant workers from three aspects: the number of close local friends, the number of local friends who can discuss important issues, and the number of local friends who can share concerns.

In order to comprehensively evaluate the urban integration of migrant workers, this paper uses a factor analysis method to reduce the dimensionality of indicators, which provides a basis for the subsequent analysis of urban integration. As shown in Table 1, firstly, the dimensionality reduction of the five indicators of economic integration is carried out, and the two common factors with eigenvalues greater than 1 are extracted by principal component analysis, the KMO value is 0.689, the Bartlett spherical test *p* value is 0.000, and the variance contribution rate is 67.30%. The value of the economic integration factor is converted into an index between 1 and 100 by a formula [44], and the higher the index, the better the economic integration. Similarly, factor analysis is performed on the indicators of social adaptation and psychological integration, and the comprehensive score is standardized as an index between 1 and 100. Finally, factor analysis is carried out on 13 indicators in three dimensions, including economic integration, social adaptation, and psychological integration, and the standardized urban integration score is obtained.

#### 3.2.2. Core Explanatory Variables

The core explanatory variable of this paper is the labor contract of migrant workers. In order to fully and accurately reflect the impact of labor contracts on migrant workers’ urban integration, this paper measures the impact of labor contracts on migrant workers’ urban integration from two perspectives: whether to sign a labor contract and the term of the labor contract. The value of the sample signing the labor contract is 1, otherwise it is 0. The term of the labor contract is subject to the actual number of years signed on the contract, and the samples without a labor contract are uniformly assigned as 0.

#### 3.2.3. Control Variables

The urban integration of migrant workers is affected by a variety of factors. Based on the previous analysis, three main control variables related to the research topic of this paper are selected, namely, human capital factors, economic factors and social capital factors. Among them, human capital factors mainly include educational background, health status, vocational skills training, and migration experience; economic factors mainly include annual household income, family land area; social capital factors mainly include number of family members, frequency of participating in social organization activities, and the nature of the unit, etc. In addition, the paper also controls the variables that may affect the urban integration of migrant workers referring to the existing research results, including gender, age, party membership, and marriage [9,31].

#### 3.2.4. Mediating Variables

The mediating variable in this paper is social security. The social security of migrant workers mainly involves social insurance, children’s education, housing and employment security [42]. The social security system in Chinese cities and towns is dominated by the social insurance system and is supplemented by the minimum living security, so the social security in this paper mainly considers social insurance. China’s current social insurance is mainly the “five social insurances and housing fund”, and by asking whether migrant workers have basic medical insurance for urban workers, basic endowment insurance for urban workers, industrial injury insurance, maternity insurance, unemployment insurance and a housing provident fund, this paper obtains the data of social insurance participation, and the positive answer is assigned as 1, otherwise it is assigned as 0. In the empirical analysis, the above six aspects are summarized to get the final social insurance participation index, with a value range of 0–6. The larger the score, the higher the social insurance participation rate of migrant workers.

The descriptive statistical results of all variables are shown in Table 2. About 47.7% of the migrant workers in the whole sample have signed a labor contract, and the average contract duration term is 1.84 years, and the proportion of migrant workers participating in social insurance is 38%. 56.2% of migrant workers are male, with an average age of about 41 years old. The education level concentrates in junior high school, 82.3% of migrant workers are married, 8.3% of migrant workers are members of the Chinese Communist Party, 83.5% of migrant workers are in good health, and 7.3% of migrant workers have participated in vocational skills training provided or subsidized by the government, while the proportion of migrant workers with more than half a year of cross-county migration experience is 25.6%. The average household population of migrant workers in the sample is four to five people, and the average family land area is 3.47 mu; the frequency of migrant workers participating in social organization activities is several times a week or daily, and 75.4% of migrant workers work in the private sector.

### 3.3. Method

#### 3.3.1. OLS Model

The OLS model is used to test the influence of a labor contract on the urban integration of migrant workers, and the empirical model is set as follows:

(1) $$Y_{i}=\alpha +\alpha_{1}X_{i}+\alpha_{2}Control_{i}+\alpha_{3}Area_{i}+\varepsilon_{i}$$

In Equation (1), the explained variable of $Y_{i}$ is the urban integration of migrant workers; the core explanatory variable of $X_{i}$ is the labor contract of migrant workers, including whether to sign a labor contract and the term of the labor contract. The control variable $Control_{i}$ is the observable personal characteristics, human capital factors, economic factors and social capital factors of the migrant workers; $Area_{i}$ is the migrant workers’ location fixed effects; and $\varepsilon_{i}$ is the error term.

#### 3.3.2. Instrumental Variable Method

Since there may be endogeneity problems between the labor contract and the urban integration of migrant workers, this paper constructs an instrumental variable model to overcome the problem of self-selection bias due to reverse causality and omitted variables, and the model is set as follows:

(2) $$X_{i}=\beta_{0}+\beta_{1}Z_{i}+\beta_{2}Control_{i}+\beta_{3}Area_{i}+\mu_{i}$$

In Equation (2), the dependent variable of $X_{i}$ is the labor contract of the migrant workers, including whether to sign labor contract and the term of the labor contract, and $Z_{i}$ is the instrumental variable. A valid instrumental variable should satisfy two conditions: first, the instrumental variable is significantly related to the labor contract; and second, the instrumental variable is exogenous. In this paper, “the average labor contract signing rate of other migrant workers in the same village” and “the average term of labor contract of other migrant workers in the same village” are used as instrumental variables.

This paper adopts the two-stage least squares (2SLS) method to correct the model estimation result and solve the problem of estimation result bias so as to obtain a consistent and unbiased estimation. The basic process of 2SLS model estimation is as follows: In the first stage, the endogenous explanatory variables (whether to sign labor contract and duration of labor contract) are used to regress the instrumental variables (average labor contract signing rate of other migrant workers in the same village and average term of labor contract of other migrant workers in the same village) to obtain the respective fittings value. In the second stage, the regressions are conducted with the explained variable (urban integration) on the fitted values in the first step. If the explanatory variable endogeneity test results reject the null hypothesis, the 2SLS model estimation results are better than the OLS estimation results.

#### 3.3.3. The Mediation Effect Model

In this paper, the stepwise test of Wen et al. [45] is used to further verify the transmission mechanism of the labor contract on migrant workers’ urban integration. The mediating effect model is set as follows:

(3) $$Y_{i}=\gamma +\gamma_{0}X_{i}+\gamma_{1}Control_{i}+\gamma_{2}Area_{i}+\sigma_{1i}$$

(4) $$M_{i}=\delta +\delta_{0}X_{i}+\delta_{1}Control_{i}+\delta_{2}Area_{i}+\sigma_{2i}$$

(5) $$Y_{i}=\lambda +\lambda_{1}X_{i}+\lambda_{2}M_{i}+\lambda_{3}Control_{i}+\lambda_{4}Area_{i}+\sigma_{3i}$$

In Equation (3) to (5), $Y_{i}$ is the urban integration of migrant workers, $X_{i}$ is the labor contract, including whether to sign a labor contract and the term of the labor contract, $M_{i}$ is the mediating variable (social security), $Control_{i}$ is the control variable, $Area_{i}$ is the area fixed effect, and $\sigma_{1i}$, $\sigma_{2i}$, $\sigma_{3i}$ are the error terms. The coefficient of $\gamma_{0}$ in Equation (3) is the total effect of labor contract on the urban integration of migrant workers. The coefficient of $\delta_{0}$ in Equation (4) is the effect of a labor contract on mediating variables. The coefficient of $\lambda_{2}$ in Equation (5) is the effect of the mediating variable on the urban integration of migrant workers after controlling for the effect of the labor contract; the coefficient of $\lambda_{1}$ is the direct effect of the labor contract on the urban integration of migrant workers after controlling for the effect of the mediating variable. In this mediating effect model, the mediating effect is the indirect effect, and the mediating effect is equal to the product of the coefficient of $\delta_{0}$ and the coefficient of $\lambda_{2}$, which is denoted as $\delta_{0}\lambda_{2}$.

Taking the social security variables of migrant workers as an example, this paper introduces the mediating effect analysis steps. The first step is to test whether the total effect of $\gamma_{0}$ of the labor contract on urban integration is significant. If it is not significant, then there is no mediating effect. If it is significant, then proceed to the next step. The second step is to test the significance of the influence coefficient of $\delta_{0}$ of the labor contract on the mediating variable (social security) and the influence coefficient of $\lambda_{2}$ of social security on the urban integration of migrant workers. If both are significant, then the mediating effect is significant, and the fourth step of inspection can be carried out. If at least one of the two is not significant, the third step of the test can be continued. The third step is to use the Bootstrap method to test whether the indirect effect is significant. If it is significant, the fourth step can be undertaken. If it is not significant, the analysis is stopped, indicating that there is no mediating effect. The fourth step is to compare the signs of $\delta_{0}\lambda_{2}$ and $\lambda_{1}$. If the signs are the same, it means that there is a mediating effect, and the weight of the mediating effect to the total effect is $\delta_{0}\lambda_{2}$/$\gamma_{0}$; if the sign is different, it means that there is a masking effect [45].

## 4. Results

### 4.1. Benchmark Regression

This paper uses the OLS model to estimate the impact of labor contracts on the urban integration of migrant workers. The results are shown in Table 3. The core explanatory variables in columns (1) to (4) are whether to sign a labor contract. The results show that the signing of labor contracts by migrant workers can significantly promote their integration into the city. Among them, signing labor contracts has a positive impact on the economic integration and the social adaptation of migrant workers, and signing labor contracts has a positive impact on psychological integration, but it is not significant. Compared with migrant workers who have not signed labor contracts, the urban integration of migrant workers who have signed labor contracts will increase by 135.4%. The core explanatory variable in columns (5) to (8) is the term of the labor contract. From the regression results, the longer the term of the labor contract, the better the urban integration of migrant workers. For each additional year of the term of the labor contract, the economic integration of migrant workers will increase by 21.9%, the social adaptation will increase by 29.3%, and the overall urban integration will increase by 20.3%. Hypothesis 1 is confirmed.

Table 3 also reflects the impact of other control variables on the urban integration of migrant workers. In terms of the effects of control variables, most of the control variables significantly affect the urban integration of migrant workers, which maintains a high consistency with the findings of previous studies. In terms of personal characteristics, male migrant workers have more advantages than female migrant workers in economic integration, but are significantly lower than females in social adaptation and psychological integration, indicating that male migrant workers are more conservative in social interaction and psychological identity. Age and party membership are negatively related to the urban integration of migrant workers, indicating that the younger the age, the higher the urban integration of migrant workers; ordinary migrant workers have a higher degree of urban integration than party-member migrant workers. Marital status has a significant impact on the economic integration, social adaptation and overall urban integration of migrant workers; and compared with unmarried migrant workers, married migrant workers have higher levels of integration in all dimensions of urban integration.

In terms of human capital factors, with the improvement of the level of education, the integration of migrant workers in terms of economic integration and social adaptation has increased significantly; but in terms of psychological aspects, it does not meant that the higher the education level is, the higher the psychological integration will be. Good health can promote the urban integration of migrant workers, further reflecting the positive impact of human capital. Migrant workers with migration experience have a higher degree of urban integration, and also show stronger integration trends in social and psychological aspects.

As for economic factors, there is a negative relationship between the land acres of migrant workers’ families and their urban integration. For every additional mu of family land, the economic integration degree of migrant workers decreased by 4.8%, the social adaptation degree decreased by 6.6%, the psychological integration degree decreased by 1.2%, and the overall urban integration degree decreased by 6.7%. Annual household income has a significant positive impact on urban integration; the higher the annual family income, the higher the economic integration, social adaptation and overall urban integration of migrant workers.

In terms of social capital factors, the number of migrant workers’ family members is significantly negatively correlated with urban integration, and the greater the number of family members, the lower the degree of urban integration of migrant workers. The higher the frequency of migrant workers participating in social organization activities, the more they can improve urban integration, but it is not statistically significant.

### 4.2. Endogeneity Discussion

The preliminary study above shows that a labor contract has a significant positive effect on the urban integration of migrant workers. In reality, therefore, there is an interaction between the labor contract and the urban integration of migrant workers, leading to endogeneity problems in the model. This paper uses an instrumental variables approach to address the endogeneity issues mentioned above, and uses “average labor contract signing rate of other migrant workers in the same village” and “average term of labor contract of other migrant workers in the same village” as the instrumental variables of whether to sign a labor contract and the term of the labor contract. The instrumental variables satisfy the requirements of relationship and exogeneity, at least theoretically. Taking the average labor contract signing rate of other migrant workers in the same village as an example, on the one hand, due to the existence of the “peer effect”, the signing of labor contracts by migrant workers from the same village will have an impact on the signing of a labor contract by other migrant workers. On the other hand, the signing of labor contracts by other migrant workers in the same village often does not directly affect the urban integration of the migrant workers. The same is true for another instrumental variable.

Columns (1) and (3) in Table 4 are the one-stage regression results of the 2SLS model. The regression results of columns (1) show that the average labor contract signing rate of other migrant workers in the village has a significant positive effect on whether the migrant worker signs the labor contract at the statistical level of 1%. In other words, the higher the average labor contract signing rate of other migrant workers in the same village of the migrant worker, the more likely the migrant worker is to sign the labor contract. The regression results of columns (3) show that the average labor contract term of other migrant workers in the same village has a significant positive impact on the labor contract term of the migrant worker at the statistical level of 1%; and the longer the average term of the labor contract of other migrant workers in the same village, the longer the term of labor contract signed by the migrant worker. The F values of columns (1) and (3) are 42.07 and 11.39, respectively, indicating that there is no weak instrumental variable.

Columns (2) and (4) in Table 4 are the two-stage regression results of the 2SLS model. The results show that after using the instrumental variable method to control the possible endogeneity of the core explanatory variables, the signing of labor contracts has a significant positive effect on the urban integration of migrant workers at the statistical level of 1%. Compared with migrant workers who have not signed labor contracts, the urban integration of migrant workers who have signed labor contracts will be about 7.4 times higher. Meanwhile, the term of the labor contract also has a significant positive effect on the urban integration of migrant workers at the statistical level of 5%. For each additional year of the term of the labor contract, the urban integration of migrant workers will increase by about 2.7 times. The above analysis shows that there is an endogeneity problem in the core explanatory variables in this paper, and after the 2SLS model is used to correct the potential endogeneity bias, the coefficient estimates of the core explanatory variables are improved. Compared with the benchmark regression, it is found that if the general OLS model is used to estimate, the positive impact of a labor contract on the urban integration of migrant workers will be underestimated due to the neglect of the endogeneity problem.

### 4.3. Robustness Check

#### 4.3.1. Excluding Sample Size

In this paper, the whole sample is randomly deleted, and the data with half of the original sample size is obtained for OLS regression. The results of column (1) in Table 5 show that whether to sign the labor contract, the term of the labor contract, and the regression conclusions of related control variables still remain robust. In addition, there are 153 migrant workers over the age of 60 in the sample used for the benchmark regression who have reached the legal retirement age and are still working. In order to further verify the robustness of the benchmark regression results, this paper excludes migrant workers over the age of 60 and re-estimates the effect of a labor contract on the urban integration of migrant workers. The regression results are shown in column (2) of Table 5. The core explanatory variables are significant and the coefficient is positive, and the labor contract still has a significant positive impact on the urban integration of migrant workers.

#### 4.3.2. Adding Control Variables

The urban integration of migrant workers may also be influenced by employment characteristics. This includes, for example, whether the unit provides housing (The value of the variable of whether the unit provides housing is as follows: for the question item “Does the unit/company you are currently working for provide housing?” If the answer is “Yes”, the variable is assigned the value of 1; if the answer is “No”, the variable is assigned a value of 0), the type of wage (The assignment method of the type of wage variable is: for the question item “What is the salary method of your current job”, if the answer is “monthly salary system” or “annual salary system”, the variable is assigned a value of 1; if the answer is “piece rate” or “hourly”, the variable is assigned a value is 0), and whether the work requires physical labor (The assignment of the heavy physical labor variable is as follows: for the question item “Do you require heavy physical labor in the course of your work”, if the answer is “never”, the variable is assigned a value of 1; if the answer is “rarely”, the variable is assigned a value of 2; if the answer is “sometimes”, the variable is assigned a value of 3; if the answer is “often”, the variable is assigned a value of 4) may affect the migrant worker’s social integration in the inflow city. Therefore, this paper adds the three control variables to Equation (1) for the robustness test, and the regression results are shown in column (3) of Table 5. The results show that a labor contract still significantly improves the urban integration of migrant workers.

### 4.4. Mechanism Test

This paper further verifies the intermediary role of social security in labor contracts and migrant workers’ urban integration, and the results are shown in Table 6. Columns (1) and (4) are benchmark regressions results, and the previous article has confirmed that labor contracts significantly affect the urban integration of migrant workers. The explained variables in columns (2) and (5) are social security. From the results, whether to sign labor contracts and the term of labor contract have a significant impact on the social security of migrant workers, indicating that signing labor contracts and increasing the term of labor contracts can both improve the social security level of migrant workers. The explained variables in columns (3) and (6) are both urban integration. The results show that the positive effect of social security on urban integration is still significant, indicating that signing labor contracts improves the participation rate of social insurance for migrant workers, enhances the social security level of migrant workers, and thus improves the urban integration of migrant workers. Social security plays a mediating role in the impact path of a labor contract on migrant workers’ urban integration, and hypothesis 2 is verified.

### 4.5. Heterogeneity Test

The previous analysis confirmed that the labor contract based on the full sample test has a significant positive impact on the urban integration of migrant workers, but did not consider the possible group heterogeneity. This paper further divides the full sample into different subsamples from the three dimensions of migrant workers’ generation, marital status and unit nature, and then examines the impact of a labor contract on the group heterogeneity of migrant workers’ urban integration. The results are shown in Table 7.

#### 4.5.1. Generation

Generation is an important level standard for dividing migrant workers group. The changes of the times have shaped the individual migrant workers, and the individual has formed distinct group attributes in the process of socialization. In this paper, migrant workers born before 1980 are defined as the older generation of migrant workers, and migrant workers born in 1980 and later are defined as the new generation of migrant workers [46]. Compared with the older generation of migrant workers, the new generation of migrant workers have obvious intergenerational differences in terms of background, growth experience, education level and life expectations [47], which will have different effects on the labor contract and the urban integration of migrant workers. The results of columns (1) and (2) in Table 7 show that the labor contract has a significant positive impact on the urban integration of the new generation of migrant workers, while the impact on the urban integration of the older generation of migrant workers is not significant. The possible explanations for this result is that the new generation of migrant workers have the basic quality and human capital rooted in the city, the labor contract signing rate is relatively high, the sense of identification with the city is relatively high, and the workers have a strong willingness to become citizens. In contrast, the older generation of migrant workers has a closer emotional connection with their hometown, a relatively low sense of identity with the city, a large gap between their lifestyles and living habits, and employment stability and social security brought by the labor contract will also attract an older generation of migrant workers to gather in cities, but the impact is relatively weak.

#### 4.5.2. Marital Status

The different marital statuses of migrant workers often leads to different urban integration. Therefore, this paper examines the heterogeneity of the impact of labor contract on urban integration of migrant workers with different marital statuses. The results of columns (3) and (4) in Table 7 show that the labor contract has a significant positive effect on the urban integration of married migrant workers, while the impact on the urban integration of unmarried migrant workers is not significant. Among married migrant workers, the urban integration of migrant workers who sign a labor contract is 1.68 times higher than that of migrant workers who do not sign a labor contract. In addition, the urban integration of married migrant workers will increase by 25.5% for each additional year of the term of the labor contract signed. The possible reason is that, compared with unmarried migrant workers, married migrant workers need to bear more family responsibilities and stable work and income. Signing labor contracts can make work more stable and income more secure; at the same time, married migrant workers think more about the offspring and hope that children can receive better education in the city. Therefore, signing labor contracts has a higher degree of integration into the city for married migrant workers.

#### 4.5.3. Unit Nature

Columns (5) and (6) in Table 7 show the impact of a labor contract on the urban integration of migrant workers in the nature of work units. The data show that the labor contract has a significant positive impact on the urban integration of migrant workers in different unit nature groups. Migrant workers working in the public sector have more higher urban integration than migrant workers working in the private sector. Signing the labor contracts in the public sector can increase the urban integration of migrant workers by about two times, while the urban integration of migrant workers in the private sector only increases by about one time. The urban integration of migrant workers in the public sector increases by 29.7% for each additional year of term of the labor contract, while the urban integration of migrant workers in the private sector increases by 16.7%. The possible reason is that the management and operation of the public sector is relatively standardized, while the management of the private sector is less standardized. Migrant workers in the public sector can enjoy more social welfare and a more of a social security system after signing the labor contract, so the degree of urban integration is higher.

## 5. Discussion

In this study, based on a large sample size survey, 1976 Chinese migrant workers were selected to study the impact of a labor contract on urban integration. This study not only focused on the impact of labor contracts on urban integration, but also investigated the impact of labor contracts of different groups on urban integration, and tested the intermediary impact of labor contracts on urban integration. We constructed a binary logit model to ensure the rationality of model selection; the instrumental variable method was used to deal with the endogeneity problem of variable selection, and the intermediary model was used as the mechanism test, which is more comprehensive. As the largest developing country with the largest number of migrant workers in the world, the research results for China have strong practical significance.

The results of the study found that the signing of labor contracts by migrant workers has a significant positive impact on their urban integration. This is consistent with the results of Wu et al. [35], who found that labor contracts can effectively promote the better integration of rural migrants into cities; by signing labor contracts, it can help rural migrants get better income and additional benefits and combat the dilemma of “unequal pay for equal work”. Ewers et al. [48] discussed the relationship between labor contracts and the well-being of migrants in the Gulf. They believed that contract-related matters were a major determinant of migrant well-being in the Gulf, and labor contracts helped improve the well-being of migrants and promote social integration. However, they mainly focused on whether the contract was honored and whether the details of employment in the contract were clear, while this paper mainly focused on the labor contract duration and whether the labor contract was signed, which leads to the difference with the results of this paper. On the other hand, the research results of Li et al. [49] support the conclusion of this study to a certain extent. Using data from 7268 questionnaires of migrant workers in Beijing, Li et al. found that labor contracts, as an institutional guarantee for migrant workers to work in Beijing, help to enhance their urban settlement. However, Li et al. only studied female migrant workers, and did not discuss the impact of male migrant workers’ labor contracts on urban integration. The results of this paper also show that there are differences within migrant workers groups, and it is because of these differences that the labor contracts of different groups of migrant workers have differences in urban integration, which shows that the new generation and married migrant workers have a higher degree of urban integration.

The effect of a labor contract on the urban integration of migrant workers makes up for the theoretical deficiencies of previous studies on the rural floating population in urban areas to a large extent. The study enriches the existing research perspective of migrant workers’ urban integration and is a beneficial expansion and supplement to the research on migrant workers’ urban integration, and provides a theoretical basis for the government to select and formulate appropriate policies for migrant workers’ urban integration. At the same time, based on the perspective of social security, this paper discusses the impact of various factors on migrant workers’ urban integration, which is helpful in understanding the mechanism of migrant workers’ urban integration.

This study has several deficiencies that can be addressed in future studies. First, it is worth noting that the 13 indicators in the three dimensions of economic integration, social adaptation and psychological integration used in this paper are not enough to comprehensively measure the urban integration of migrant workers, and the existing literature cannot fully make up for this deficiency. How to establish a comprehensive and reasonable index to measure the urban integration of migrant workers is worthy of further study in the future. Second, we selected the cross-sectional data of 2018 CLDS as the research sample for this paper. However, the influence of labor contracts on the urban integration of migrant workers is a dynamic process. Thus, future research could use panel data to further expend and verify the relationship in greater detail. Third, studying the relationship between labor contracts and migrant workers’ urban integration also involves many missing variables, such as cultural and entertainment expenditure, dialect distance, left-behind experience and mental health, etc. [18,46]. If these variables can be controlled, the processing effect will be cleaner, but it is beyond the scope of this article to discuss them due to the availability of data and samples. Finally, since the data of this study come from a field survey in 2018, the results are only for the situation at that time. As the signing of labor contracts of migrant workers has received widespread attention from all walks of life in recent years, the social policies and environment for protecting the rights and interests of migrant workers have been improving, and the level of well-being of migrant workers is gradually rising. Therefore, the conclusions of this paper should be interpreted from a development perspective and a cautious attitude.

## 6. Conclusions

This paper studies the urban integration of migrant workers, aiming to examine the relationship between labor contracts and the urban integration of migrant workers. This paper constructs a measurement system of migrant workers’ urban integration, theoretically analyzes the internal mechanism of the labor contract on migrant workers’ urban integration, and empirically tests the impact of a labor contract on migrant workers’ urban integration and its mechanism based on the 2018 data of the China Labor Dynamics Survey. Generally speaking, the main content of this paper can be divided into four parts. First of all, according to the theory of social integration and social security, it was found that signing labor contracts is conducive to promoting the urban integration of migrant workers. Secondly, based on the OLS model, the effects of signing labor contracts and the term of the labor contract on the economic integration, social adaptation, psychological integration and overall urban integration of migrant workers were investigated. At the same time, in order to solve the endogenous problem of labor contracts, this paper selects “the average labor contract signing rate of other migrant workers in villages” and “the average labor contract duration of other migrant workers in villages” as instrumental variables, and uses the two-stage least squares method to test them. Thirdly, from the perspective of social security, this paper proposes the internal mechanism of the role of labor contracts on migrant workers’ urban integration, and conducts an empirical test using the mediation effect model. Finally, the heterogeneity is analyzed in terms of age, marital status and unit nature. The main conclusions of this paper are as follows:

First, this study shows that signing the labor contracts and the term of the labor contract is significantly and positively correlated with the urban integration of migrant workers. Considering the possible endogeneity bias, the basic conclusion of this study is still supported after further using the 2SLS model analysis and robustness test. Second, the study confirms that labor contracts can significantly improve the social security level of migrant workers, and can promote the urban integration of migrant workers through the mediating effect of social security. Finally, a further heterogeneity analysis shows that compared with the older generation, unmarried and private sector migrant workers, the labor contract of the new generation, and married migrant and public sector migrant workers have a significant positive impact on urban integration.

Based on the above findings, the following insights can be stated: First, the urban integration degree of migrant workers who sign labor contracts is significantly higher than that of migrant workers who do not sign labor contracts. Since the implementation of the labor contract law, the signing rate of labor contracts for migrant workers has increased to a certain extent, but the overall rate is still low. The government should strengthen labor market supervision and urge employers to implement the Labor Contract Law, which will contribute to improving the signing rate of labor contracts for migrant workers, protect the legitimate rights and interests of migrant workers, and promote the urban integration of migrant workers. Second, the government should encourage employers to sign long-term labor contracts with migrant workers, which is conducive to stabilizing the employment of migrant workers and promoting the integration of migrant workers into cities. Third, the government should strengthen the construction of the social security system for migrant workers, gradually incorporate qualified migrant workers into the social security system for urban residents, and focus on solving problems such as housing security, social insurance and children’s education for migrant workers.

## Figures and Tables

**Table 1:** Indicator description of urban integration and factor analysis.

Dimension	Indicators	Indicator Description	Factor Analysis
Economic integration	Salary income	Average monthly wage income of migrant workers in 2017 (logarithm)	KMO: 0.689 Bartlett: *p* < 0.01 Eigenvalue > 1.0 extracts two common factors Cumulative variance contribution rate: 67.30%
	Job satisfaction	1 = very satisfied; 2 = satisfied; 3 = in general; 4 = dissatisfied; 5 = very dissatisfied	
	Satisfaction with respect for work	1 = very satisfied; 2 = satisfied; 3 = in general; 4 = dissatisfied; 5 = very dissatisfied	
	Work environment satisfaction	1 = very satisfied; 2 = satisfied; 3 = in general; 4 = dissatisfied; 5 = very dissatisfied	
	Household consumption expenditure	Average monthly consumption expenditure of migrant workers’ households in 2017 (logarithm)	
Psychological integration	Number of close local friends	Actual number of people	KMO: 0.637 Bartlett: *p* < 0.01 Eigenvalue > 1.0 extracts a common factor Cumulative variance contribution rate: 72.73%
	Number of local friends who can discuss important issues	Actual number of people	
	Number of local friends who can share concerns	Actual number of people	
Social adaptation	Familiarity with local residents	1 = very familiar; 2 = familiar; 3 = in general; 4 = unfamiliar; 5 = very unfamiliar	KMO: 0.750 Bartlett: *p* < 0.01 Eigenvalue > 0.9 extracts two common factors Cumulative variance contribution rate: 68.20%
	Mutual assistance with local residents	1 = very much; 2 = much; 3 = in general; 4 = relatively little; 5 = very little	
	Level of trust in local residents	1 = very trusting; 2 = trusting; 3 = in general; 4 = distrustful; 5 = very distrustful	
	Participating in village/neighborhood committee elections	1 = yes; 0 = no	
	Local life happiness	1 = very unhappy; 2 = unhappy; 3 = in general; 4 = happy; 5 = very happy	
Urban integration	13 indicators of three dimensions: economic integration, social adaptation and psychological integration		KMO: 0.739 Bartlett: *p* < 0.01 Eigenvalue > 1.0 extracts four common factors Cumulative variance contribution rate: 62.77%

Note: Data source CLDS2018, observed value 1976.

**Table 2:** Meaning of variables and descriptive statistics.

Variable Type	Variable Name	Variable Meaning and Assignment	Average Value	Standard Deviation
Explained variables	Urban integration	Factor values	30.579	10.327
	Economic integration	Factor values	46.575	13.733
	Social adaptation	Factor values	49.219	15.046
	Psychological integration	Factor values	31.787	3.837
Core explanatory variables	Whether to sign labor contract	1 = yes; 0 = no	0.477	0.500
	Term of labor contract	Actual term of the contract (years)	1.840	3.070
Intermediate variables	Social security	Social insurance participation index: actual values	1.326	2.023
Personal characteristics	Gender	1 = male; 0 = female	0.562	0.496
	Age	Actual age of migrant workers (years)	41.124	12.303
	Marriage	1 = married; 0 = unmarried	0.826	0.379
	Party membership	1 = CPC member; 0 = others	0.083	0.276
Human capital factors	Educational background	1 = primary school and below; 2 = junior high school; 3 = high school, junior college; 4 = college and above	2.334	1.007
	Health status	1 = very healthy; 2 = healthy; 3 = general 4 = unhealthy; 5 = very unhealthy	2.088	0.835
	Vocational skills training	Whether migrant workers have participated in vocational skills training: 1 = yes; 0 = no	0.073	0.260
	Migration experience	Whether migrant workers have more than half a year of cross-county migration experience: 1 = yes; 0 = no	0.256	0.436
Economic factors	Family land area	Land area of migrant workers’ households (mu)	3.466	8.640
	Annual household income	Actual income (logarithm)	10.927	1.126
Social capital factors	Number of family members	Actual population (persons)	4.661	2.038
	Participating in social organization activities	1 = never; 2 = several times a year or less; 3 = several times a month; 4 = several times a week; 5 = daily	4.718	0.664
	Nature of the unit	1 = public sector; 0 = private sector	0.246	0.431

Note: Data source CLDS2018, observed value 1976.

**Table 3:** Regression results of the impact of labor contract on the urban integration of migrant workers.

Variable Name	Economic Integration (1)	Social Adaptation (2)	Psychological Integration (3)	Urban Integration (4)	Economic Integration (5)	Social Adaptation (6)	Psychological Integration (7)	Urban Integration (8)
Whether to sign labor contract	1.644 ** (0.640)	1.267 * (0.689)	0.013 (0.207)	1.354 *** (0.474)				
Term of labor contract					0.219 ** (0.097)	0.293 *** (0.102)	−0.005 (0.027)	0.203 *** (0.077)
Gender	2.795 *** (0.609)	−1.092 * (0.647)	−0.366 ** (0.173)	1.416 *** (0.443)	2.772 *** (0.610)	−1.136 * (0.646)	−0.365 ** (0.173)	1.393 *** (0.444)
Age	−0.217 *** (0.032)	−0.315 *** (0.035)	−0.049 *** (0.008)	−0.152 *** (0.025)	−0.222 *** (0.032)	−0.316 *** (0.034)	−0.049 *** (0.008)	−0.156 *** (0.025)
Marital status	4.604 *** (0.939)	1.694 * (0.959)	0.282 (0.254)	1.968 *** (0.665)	4.605 *** (0.938)	1.613 * (0.953)	0.286 (0.251)	1.954 *** (0.664)
Party membership	−2.256 ** (1.141)	−3.320 *** (1.168)	−1.002 *** (0.270)	−2.266 ** (0.892)	−2.412 ** (1.136)	−3.445 *** (1.166)	−1.003 *** (0.269)	−2.395 *** (0.889)
Educational background	0.175 (0.356)	0.519 (0.389)	−0.039 (0.114)	0.549 * (0.286)	0.234 (0.356)	0.494 (0.387)	−0.035 (0.110)	0.584 ** (0.282)
Health status	2.162 *** (0.386)	0.630 (0.397)	0.609 *** (0.109)	1.403 *** (0.271)	2.153 *** (0.385)	0.624 (0.396)	0.609 *** (0.108)	1.396 *** (0.271)
Vocational skills training	−3.439 *** (1.118)	−2.015 (1.308)	−0.652 ** (0.291)	−1.853 ** (0.908)	−3.415 *** (1.117)	−2.003 (1.305)	−0.651 ** (0.291)	−1.834 ** (0.905)
Migration experience	0.131 (0.672)	7.031 *** (0.775)	0.948 *** (0.195)	2.702 *** (0.501)	0.168 (0.670)	7.000 *** (0.775)	0.951 *** (0.197)	2.721 *** (0.500)
Number of mu of family land	−0.048 ** (0.020)	−0.066 *** (0.025)	−0.012 ** (0.005)	−0.067 *** (0.015)	−0.050 *** (0.019)	−0.066 *** (0.025)	−0.012 ** (0.005)	−0.068 *** (0.015)
Annual household income	1.252 *** (0.364)	0.769 ** (0.317)	−0.056 (0.058)	0.893 *** (0.210)	1.279 *** (0.363)	0.783 ** (0.318)	−0.055 (0.058)	0.914 *** (0.211)
Number of family members	−0.227 (0.146)	−0.201 (0.157)	−0.058 * (0.035)	−0.328 *** (0.105)	−0.229 (0.146)	−0.188 (0.158)	−0.059 * (0.035)	−0.326 *** (0.104)
Participating in social organization activities	0.358 (0.456)	0.597 (0.468)	0.170 (0.109)	0.360 (0.333)	0.348 (0.456)	0.590 (0.467)	0.170 (0.109)	0.352 (0.332)
Nature of the unit	−2.901 *** (0.778)	−1.318 * (0.793)	−0.417 * (0.217)	−1.358 ** (0.630)	−2.760 *** (0.778)	−1.270 (0.788)	−0.412 * (0.220)	−1.252 ** (0.630)
Regional variables	Controlled	Controlled	Controlled	Controlled	Controlled	Controlled	Controlled	Controlled
Constant term	31.426 *** (5.010)	47.248 *** (4.651)	32.638 *** (0.907)	19.691 *** (3.176)	31.687 *** (5.003)	47.382 *** (4.657)	32.644 *** (0.910)	19.894 *** (3.186)
R^2^	0.096	0.159	0.078	0.115	0.095	0.161	0.078	0.114
Observations	1976	1976	1976	1976	1976	1976	1976	1976

Note: ***, **, * denote significance at the 1%, 5%, and 10% levels, respectively.

**Table 4:** 2SLS estimation results of labor contract and urban integration of migrant workers.

Variable Name	Whether to Sign Labor Contract (1)	Urban Integration (2)	Term of Labor Contract (3)	Urban Integration (4)
Whether to sign labor contract		7.419 *** (1.942)		
Term of labor contract				2.760 ** (1.238)
Average labor contract signing rate of other migrant workers in the same village	0.427 *** (0.038)			
Average term of labor contract of other migrant workers in the same village			0.191 *** (0.057)	
Control variables	Controlled	Controlled	Controlled	Controlled
Regional variables	Controlled	Controlled	Controlled	Controlled
Constant term	0.143 (0.154)	18.711 *** (3.267)	0.392 (1.000)	18.629 *** (4.105)
F-value	42.07		11.39	
R^2^	0.205		0.082	
Observations	1976	1976	1976	1976

Note: ***, ** represent the significance level at 1%, 5% respectively; robust standard error in parentheses.

**Table 5:** Robustness test of the impact of the labor contract on the urban integration of migrant workers.

Variable Name	Randomly Censoring Subsamples (1)		Excluding Samples Over 60 Years Old (2)		Adding Control Variables (3)	
Whether to sign labor contract	1.924 *** (0.643)		1.449 *** (0.485)		1.539 *** (0.482)	
Term of labor contract		0.381 *** (0.092)		0.228 *** (0.077)		0.216 *** (0.077)
Whether the unit provides housing					−0.776 (0.501)	−0.640 (0.498)
Wage type					0.073 (0.509)	0.171 (0.509)
Heavy physical work					0.662 *** (0.222)	0.654 *** (0.222)
Control variables	Controlled	Controlled	Controlled	Controlled	Controlled	Controlled
Regional variables	Controlled	Controlled	Controlled	Controlled	Controlled	Controlled
Constant term	19.481 *** (4.109)	19.973 *** (4.075)	19.188 *** (3.246)	19.423 *** (3.258)	17.894 *** (3.244)	18.068 *** (3.251)
R^2^	0.144	0.148	0.102	0.102	0.120	0.120
Observations	988	988	1823	1823	1976	1976

Note: *** denote significant at the 1% levels.

**Table 6:** Test of the mediating effect of labor contract and urban integration of migrant workers.

Variable Name	Urban Integration (1)	Social Security (2)	Urban Integration (3)	Urban Integration (4)	Social Security (5)	Urban Integration (6)
Whether to sign labor contract	1.354 *** (0.474)	1.579 *** (0.082)	0.406 (0.526)			
Term of labor contract				0.203 *** (0.077)	0.184 *** (0.017)	0.093 (0.083)
Social security			0.600 *** (0.136)			0.596 *** (0.132)
Control variables	Controlled	Controlled	Controlled	Controlled	Controlled	Controlled
Regional variables	Controlled	Controlled	Controlled	Controlled	Controlled	Controlled
Constant term	19.691 *** (3.176)	−0.351 (0.562)	19.901 *** (3.128)	19.894 *** (3.186)	−0.085 (0.573)	19.945 *** (3.131)
R^2^	0.115	0.368	0.123	0.114	0.311	0.124
Observations	1976	1976	1976	1976	1976	1976

Note: *** denote significant at the 1% levels.

**Table 7:** Heterogeneous effects of the labor contract on the urban integration of migrant workers.

Variable Name	Generation		Marital Status		Unit Nature	
	Older Generation (1)	New Generation (2)	Married (3)	Unmarried (4)	Public Sector (5)	Private Sector (6)
Panel: A						
Whether to sign labor contract	1.091 (0.665)	1.452 ** (0.701)	1.317 ** (0.529)	1.681 (1.078)	2.063 * (1.098)	1.073 ** (0.533)
Control variables	Controlled	Controlled	Controlled	Controlled	Controlled	Controlled
Regional Variables	Controlled	Controlled	Controlled	Controlled	Controlled	Controlled
R^2^	0.097	0.110	0.117	0.137	0.117	0.109
Observations	1098	878	1633	343	487	1489
Panel: B						
Term of labor contract	0.142 (0.107)	0.243 ** (0.113)	0.195 ** (0.087)	0.245 (0.162)	0.297 ** (0.140)	0.167 * (0.094)
Control variables	Controlled	Controlled	Controlled	Controlled	Controlled	Controlled
Regional variables	Controlled	Controlled	Controlled	Controlled	Controlled	Controlled
R^2^	0.097	0.111	0.117	0.137	0.117	0.109
Observations	1098	878	1633	343	487	1489

Note: **, * denote significant at the 5% and 10% levels, respectively.

## Data Availability

The data presented in this study are available on request from the corresponding author.

## References

[1] Lin M., Yang T. (2020). Geography, trade, and internal migration in China. J. Urban Econ..

[2] Zhou J.J., Zhu L., Zhang J.W. (2022). Social Integration and Health Among Young Migrants in China: Mediated by Social Mentality and Moderated by Gender. Front. Psychol..

[3] (2021). National Bureau of Statistics in China. http://www.stats.gov.cn/english/.

[4] Wu S.L. (2016). The contribution of agricultural labor migration to economic growth in China. Econ. Res. J..

[5] Liang Z. (2016). China’s great migration and the prospects of a more integrated society. Rev. Sociol..

[6] Fan C.C. (2004). The state, the migrant labor regime, and maiden workers in China. Polit. Geogr..

[7] Knight J., Song L. (1999). The Rural-Urban Divide Economic Disparities and Interactions in China.

[8] Zhang K.H., Song S. (2003). Rural-urban migration and urbanization in China: Evidence from time-series and cross-section analyses. China Econ. Rev..

[9] Huang X., Liu Y., Xue D., Li Z., Shi Z. (2018). The effects of social ties on rural-urban migrants’ intention to settle in cities in China. Cities.

[10] Huang G., Zhang H., Xue D. (2018). Beyond unemployment: Informal employment and heterogeneous motivations for participating in street vending in present-day China. Urban Stud..

[11] Li X.Y., Richard B.F. (2015). How does China’s New Labour Contract Law affect floating workers?. Br. J. Ind. Relat..

[12] Yang J. (2013). Social exclusion and young rural-urban migrants’ integration into a host society in China. Ann. Am. Acad. Pol. Soc. Sci..

[13] Ruiz-Tagle J. (2013). A theory of socio-spatial integration: Problems, policies and concepts from a U.S. perspective. Int. J. Urban Reg. Res..

[14] Gordon M.M. (1964). Assimilation in American Life: The Role of Race.

[15] Junger-Tas J. (2001). Ethnic minorities, social integration and crime. Eur. J. Crim. Pol. Res..

[16] Entzinger H., Renske B. (2003). Benchmarking in Immigrant Integration.

[17] Yang J.H. (2009). From isolation, selective integration to integration: Theoretical reflections on the social integration of floating populations. Popul. Stud..

[18] Wang G.X., Shen J.F., Liu J.B. (2008). Citizenship of peasant migrant during urbanization in China: A case study of Shanghai. Popul. Dev..

[19] Zhang C., Ma B., Qiu H.G. (2022). Can the use of ICT improve the social integration of displaced households in poverty alleviation resettlement program?. Chin. Rural. Econ..

[20] Qiu H., Zhou W.J. (2019). A Study on migrants’ settling willingness and its influencing factors. Popul. J..

[21] Yang G., Zhou C.S., Jin W.F. (2020). Integration of migrant workers: Differentiation among three rural migrant enclaves in Shenzhen. Cities.

[22] Wang Z.C., Liu J.C., Ming J. (2020). Owned a house in an urban destination or made housing investments in the hometown? Determinants of rural migrants’ housing attainments in China. Hous. Policy Debate.

[23] Chen H.S., Wang X.P., Liu Y., Liu Y.Q. (2020). Migrants’ choice of household split or reunion in China’s urbanisation process: The effect of objective and subjective socioeconomic status. Cities.

[24] Xiao Y., Miao S.Q., Sarkar C., Fan L.Y., Li Z.G. (2020). Do neighborhood ties matter for residents’ mental health in affordable housing: Evidence from Guangzhou, China. Cities.

[25] Han C. (2015). Explaining the subjective well-being of urban and rural Chinese: Income, personal concerns, and societal evaluations. Soc. Sci. Res..

[26] Ye J.Z. (2017). Stayers in China’s “hollowed-out” villages: A counter narrative on massive rural-urban migration. Popul. Space Place.

[27] Angelo M., Antonio P. (2017). Dealing with omitted answers in a survey on social integration of immigrants in Italy. Math. Popul. Stud..

[28] Takeda S., Arimura T.H., Sugino M. (2019). Labor market distortions and welfare-decreasing international emissions trading. Environ. Resour. Econ..

[29] Massey D.S., Arango J., Hugo G., Kouaouci A., Pellegrino A., Taylor J.E. (1993). Theories of international migration: A review and appraisal. Pop. Dev. Rev..

[30] Meng X., Xue S. (2020). Social networks and mental health outcomes: Chinese rural-urban migrant experience. J. Popul. Econ..

[31] Chen J., Wang W. (2019). Economic incentives and settlement intentions of rural migrants: Evidence from China. J. Urban Aff..

[32] Lin S.N., Gaubatz P. (2017). Socio-spatial segregation in China and migrants’ everyday life experiences: The case of Wenzhou. Urban Geogr..

[33] Ouyang W., Wang B.Y., Tian L., Niu X.Y. (2017). Spatial deprivation of urban public services in migrant enclaves under the context of a rapidly urbanizing China: An evaluation based on suburban Shanghai. Cities.

[34] Meng L., Zhao M.Q. (2018). Permanent and temporary rural-urban migration in China: Evidence from field surveys. China Econ. Rev..

[35] Wu Y.L., Xiao H. (2021). The Labor Contract Law and the economic integration of rural migrants in urban China. Asian Pac. Migr. J..

[36] Arif I. (2020). The determinants of international migration: Unbundling the role of economic, political, and social institutions. World Econ..

[37] Liu L., Huang Y.Q., Zhang W.H. (2018). Residential segregation and perceptions of social integration in Shanghai, China. Urban Stud..

[38] Hau B., Nazroo J., Banks J., Marshall A. (2019). Impacts of migration on health and well-being in later life in China: Evidence from the China Health and Retirement Longitudinal Study (CHARLS). Health Place.

[39] Zhang Y., Wang H. (2011). Household and geographical discrimination in urban labor market: A study based on census data. Manag. World.

[40] Huang X., Dijst M., Van W.J., Zou N.J. (2014). Residential mobility in China: Home ownership among rural-Urban migrants after reform of the hukou registration system. J. Hous. Built. Environ..

[41] Gao Q., Yang S., Li S. (2012). Labor contracts and social insurance participation among migrant workers in China. China Econ. Rev..

[42] Du P.C., Xu S., Wu M.Q. (2018). Labor protection and welfare improvement for rural migrant workers: The new Labor Contract Law perspective. Econ. Res..

[43] Hamermesh D.S., Trejo S.J. (2013). How do immigrants spend their time? The process of assimilation. J. Popul. Econ..

[44] Nie W., Feng X.T. (2016). Employment quality, social interaction and migrant workers’ willingness to enter households: Based on the survey of migrant workers in the Pearl River Delta and Yangtze River Delta. Agric. Econ. Issues.

[45] Wen Z.L., Ye B.J. (2014). Analysis of mediating effects: The development of methods and model. Adv. Psychol. Sci..

[46] Liu J.F., Wei H.K. (2022). How does dialect distance affect permanent migration intension of migrant workers: From the perspective of social integration. China Rural. Obs..

[47] Sakdapolrak P., Naruchaikusol S., Ober K. (2016). Migration in a changing climate: Towards a translocal social resilience approach. DIE ERDE–J. Geogr. Soc. Berl..

[48] Ewers M.C., Diop A., Le K.T., Bader L. (2020). Migrant worker well-being and its determinants: The case of Qatar. Soc. Indic. Res..

[49] Li Y.C. (2016). Influence factor analysis of female migrant workers’ willingness to reside in cities: A case study of Beijing. J. Hunan Univ. Sci. Technol. (Soc. Sci. Ed.).

